# DNA-induced 2′3′-cGAMP enhances haplotype-specific human STING cleavage by dengue protease

**DOI:** 10.1073/pnas.1922243117

**Published:** 2020-06-23

**Authors:** Chan-I Su, Yu-Ting Kao, Chao-Chen Chang, Yao Chang, Tzong-Shiann Ho, H. Sunny Sun, Yi-Ling Lin, Michael M. C. Lai, Yu-Huei Liu, Chia-Yi Yu

**Affiliations:** ^a^National Institute of Infectious Diseases and Vaccinology, National Health Research Institutes, Miaoli 350, Taiwan;; ^b^Department of Microbiology and Immunology, National Cheng Kung University, 701 Tainan, Taiwan;; ^c^Department of Pediatrics, National Cheng Kung University, 701 Tainan, Taiwan;; ^d^Institute of Molecular Medicine, National Cheng Kung University, 701 Tainan, Taiwan;; ^e^Institute of Biomedical Sciences, Academia Sinica, 115 Taipei, Taiwan;; ^f^Research Center for Emerging Viruses, China Medical University Hospital, 404 Taichung, Taiwan;; ^g^Institute of Molecular Biology, Academia Sinica, 115 Taipei, Taiwan;; ^h^Graduate Institute of Integrated Medicine, China Medical University, 404 Taichung, Taiwan

**Keywords:** STING, DENV protease, SNP, 2′3′-cGAMP, cGAS

## Abstract

The cytosolic DNA sensor cGMP-AMP synthase (cGAS) synthesizes the noncanonical cyclic dinucleotide 2′3′-cGAMP to activate the adaptor protein stimulator of IFN genes (STING), thus awakening host immunity in response to DNA pathogen infection. However, dengue virus (DENV), an RNA virus without a DNA stage in its life cycle, also manipulates cGAS-STING–mediated innate immunity by proteolytic degradation of STING. Here, we found that the sensitivity of STING to DENV protease varied with different human STING haplotypes. Exogenous DNA further enhanced DENV protease’s ability to interact and cleave protease-sensitive STING. DNA-enhanced STING cleavage was reduced in cGAS-knockdown cells and triggered by the cGAS product 2′3′-cGAMP. The source of DNA may not be endogenous mitochondrial DNA but rather exogenous reactivated viral DNA. Cells producing 2′3′-cGAMP by overexpressing cGAS or with DNA virus reactivation enhanced STING cleavage in neighboring cells harboring DENV protease. DENV infection reduced host innate immunity in cells with the protease-sensitive STING haplotype, whose homozygote genotype frequency was found significantly reduced in Taiwanese people with dengue fever. Therefore, the human STING genetic background and DNA pathogen coinfection may be the missing links contributing to DENV pathogenesis.

## Significance

Dengue virus (DENV) antagonizes the DNA sensing cGAS-STING pathway to subvert innate immunity, but how DENV protease-mediated human STING cleavage contributes to DENV pathogenesis remains obscure. Here, we found that STING haplotype frequency varies among different subhuman populations, and different haplotypes respond differently to DENV protease. The cleavage of a DENV protease-sensitive STING can be further enhanced by coculture with neighboring cells producing 2′3′-cGAMP, either by DNA transfection of cGAS or by reactivating Epstein–Barr virus from latent infection. Thus, DENV infection trims down human STING-mediated innate immunity in a haplotype-specific manner. The genetic background of host STING and bystander coinfection of pathogens triggering 2′3′-cGAMP production may be the missing link between STING cleavage and DENV pathogenesis.

Dengue virus (DENV) infection threatens millions of people annually, but effective therapeutics and prognostic markers for this disease are still lacking. Single-nucleotide polymorphisms (SNPs) have been associated with many genetic disorders, cancers, and infectious diseases. The SNPs in immunity-related host genes may contribute to the progression of both inflammatory and infectious disease (1). The SNP rs12979860 near the IL28B gene has been associated with the responsiveness of hepatitis C patients to interferon (IFN)-based therapy (2). For DENV infection, a variant in the promoter region of the DENV receptor DC-SIGN/CD209 and the pattern recognition receptor (PRR) Toll-like receptor 4 (TLR4) haplotypes have been found associated with severity of dengue disease (3, 4). However, DC-SIGN/CD209 is one of the multiple DENV receptors identified (5), and TLR4 is not a DENV-specific PRR. Although DENV protease specifically cleaves human stimulator of IFN genes (STING) (6–8) to subvert innate immunity, whether the human STING variants affect this cleavage event or contribute to DENV pathogenesis remains to be uncovered.

As the first line of host defense, innate immunity identifies pathogens via PRRs to induce cytokines and regulate the adaptive immune system. DENV infection could trigger the production of antiviral IFN by activating PRR-sensing foreign RNA. To complete virus replication, DENV evolves multiple strategies to counteract host antiviral responses (9). Despite lack of a DNA stage in DENV replication, DENV targets the cGAS-STING–mediated DNA-sensing pathway (6, 7, 10). The K27-linked polyubiquitination of DENV NS3 protein enhances the formation of the NS2B3 protease complex and, thus, contributes to STING cleavage (11). The sole DENV protease NS2B3 explicitly cleaves human STING but not its murine ortholog, which suggests different DENV restriction factors among different species (6, 7). Moreover, the failure of DENV protease in cleaving nonhuman primate STINGs derived from presumed DENV natural reservoirs (8) indicates the DENV-induced STING cleavage might be the factor contributing to species-specific pathogenesis of DENV infection.

cGMP-AMP synthase (cGAS) catalyzes the synthesis of a noncanonical cyclic dinucleotide (CDN) 2′3′-cGAMP in response to DNA stimuli present in cytoplasm (12, 13). cGAS-produced 2′3′-cGAMP, which can be transferred to neighboring cells (14), serves as a second messenger binding to the adaptor protein STING to induce IFN (15). Thus, the cGAS enzyme-dead GS/AA mutation is inert in producing 2′3′-cGAMP and triggering IFN production (16). The activated STING can be further regulated posttranslationally via specific phosphorylation and ubiquitination (17). More than 12 human STING haplotypes in nature have been identified (18), but the vulnerability of human STING to DENV protease remains unclear.

## Results

### Top Three Human STING Haplotypes Respond Differently to Dengue Protease Cleavage.

**Fig. 1.**
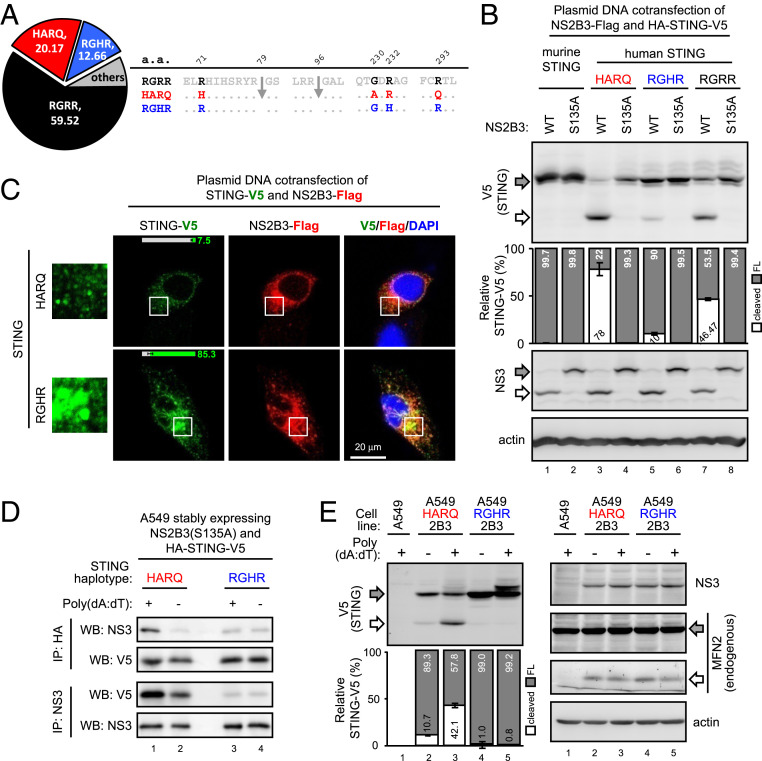
DENV protease-mediated STING cleavage varies by STING haplotype. (*A*) The nomenclature and frequency (%) of human STING haplotypes in this study. The haplotype frequency (% in pie chart) was analyzed by the web-based application LDlink with the NCBI 1000 Genomes Project data. Arrow, the putative dengue protease cleavage sites mapped previously (6–8). (*B*) A549 cells were cotransfected with DENV protease NS2B3 and each STING for 18 h, then examined by Western blot (WB) analysis with the indicated primary antibodies. S135A, protease-dead NS2B3 mutant. Gray arrow, full-length STING-V5 or NS2B3; white arrow, cleaved product of STING or NS3. (*C*) A549 cells were cotransfected with DENV NS2B3 and the indicated STING haplotype overnight, then analyzed by IFA with the indicated antibodies. The NS2B3-harboring cells displaying STING in cytoplasmic puncta were quantified and embedded as percentage bars (mean ± SD, *n* = 3 per group). The confocal images were taken by Olympus FV1000 (1,600 × 1,600 pixels, gain = 1–3×). (*D*) A549 cells stably coexpressing protease-dead NS2B3 and the indicated STING in the presence (+) or absence (-) of poly(dA:dT) stimuli were analyzed by coimmunoprecipitation and WB. (*E*) A549 cells stably coexpressing DENV protease (2B3) and each STING were stimulated with the synthetic analog of B-form DNA poly(dA:dT) (1 μg/mL) for 4 h, then analyzed by WB with the indicated antibodies. Gray arrow, full-length STING-V5 or endogenous MFN2; white arrow, cleaved STING or MFN2. Quantification of the STING cleavage levels was in a bar graph. The full-length (FL; gray bar) and cleaved (white bar) STING-V5 signal were divided by the total STING-V5 signal of each lane for the relative ratio in percentage. Data are mean ± SD (*n* = 3 per group).

Among more than 650 SNPs and 12 major STING haplotypes in the human population, four missense variations at residues 71, 230, 232, and 293 determine the top three haplotypes, RGRR, HARQ, and RGHR, covering >90% of the human population worldwide (Fig. 1*A*). We cloned each of the three STINGs with the HARQ (7) backbone (*SI Appendix*, Fig. S1) and cotransfected them with DENV protease NS2B3 to test its sensitivity to DENV protease. Various levels of cleaved STING products were detected on human but not murine STING when cotransfected with WT but not the enzyme-dead S135A mutated protease (Fig. 1*B*). Among the three human STINGs, HARQ and RGHR were the most sensitive and resistant haplotype to NS2B3, respectively (Fig. 1*B*). RGRR haplotype was not included in the following study because of its moderate cleavage efficiency. While the STINGs were cleaved with different efficiency, the DENV protease-mediated cleavage of mitofusin 2 (MFN2) (19) is not altered (*SI Appendix*, Fig. S2), suggesting the haplotype of STING, rather than the protease activity of NS2B3, affects the STING cleavage here. Immunofluorescence assay (IFA) revealed more substantial STING activation aggregates (20) for the protease-resistant RGHR than protease-sensitive HARQ haplotype on cotransfection with DENV protease (Fig. 1*C*, the green signal in white square enlarged at *Left*).

Because none of the four SNP residues located at the putative cleavage sites of DENV protease (Fig. 1*A*), we checked their interactions to understand the mechanism contributing to the different cleavage efficiency. Both STINGs coimmunoprecipitated with DENV protease, but more pull-down was noted with sensitive HARQ than resistant RGHR (*SI Appendix*, Fig. S3), which suggests that the higher level of STING cleavage might result from a stronger interaction between DENV protease and STING.

Because the cotransfection experiment provided DENV protease and STING with exogenous DNA plasmids (Fig. 1 *B* and *C* and *SI Appendix*, Figs. S2 and S3), we asked whether DNA stimulation contributes to the binding activity and cleavage efficiency of different STINGs. Cell lines stably coexpressing each STING and NS2B3 (WT and enzyme-dead S135A) were established and stimulated with poly(dA:dT) transfection. The DNA stimulation enhanced the interaction between DENV protease and sensitive STING HARQ but not resistant RGHR (Fig. 1*D*). Furthermore, the poly(dA:dT)-enhanced interaction increased the cleavage of the sensitive HARQ but not resistant RGHR haplotype (Fig. 1*E*, lanes 2–3 vs. lanes 4–5). To clarify the type of DNA contributing to this cleavage event, various DNA species were prepared (*SI Appendix*, Fig. S4 *A* and *B*) to stimulate human A549 cells stably coexpressing DENV protease NS2B3 and each STING (A549-HARQ-2B3 and A549-RGHR-2B3). Accordingly, all DNA tested enhanced DENV protease-mediated STING cleavage of the sensitive HARQ but not resistant RGHR haplotype (*SI Appendix*, Fig. S4*C*). The DNA-induced posttranslational modification of STING (17) (*SI Appendix*, Fig. S4*C*, indicated by asterisk) in A549-RGHR-2B3 was attenuated in A549-HARQ-2B3, which further supports that DENV protease functionally suppresses STING by cleavage in a haplotype-specific manner.

### Endogenous cGAS but Not Mitochondrial DNA Is Indispensible in Enhancing the STING Cleavage Event.

**Fig. 2.**
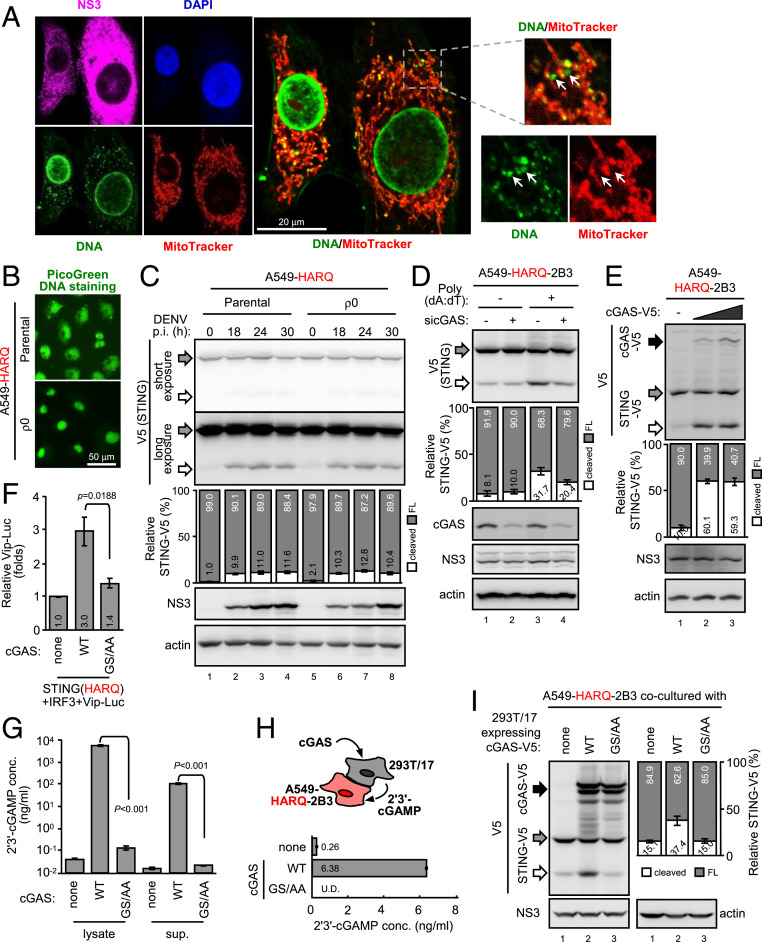
cGAS but not mtDNA mediates the enhanced STING cleavage of HARQ haplotype. (*A*) A549 cells were infected with DENV (multiplicity of infection [moi] 10) for 48 h, then fixed for IFA. Green, anti-DNA; red, MitoTracker; magenta, anti-DENV NS3; blue, DAPI nuclear counterstaining. The confocal images were taken by Olympus FV1000 (1,600 × 1,600 pixels, gain = 1–3×). (*B*) mtDNA-depleted A549-HARQ-ρ0 and its parental cells were live stained with PicoGreen DNA dye. (*C*) A549-HARQ and its ρ0 cells were infected with DENV (moi 5) and harvested at the indicated time for WB analysis. p.i., postinfection. (*D*) A549 cells stably coexpressing STING HARQ and DENV protease (A549-HARQ-2B3) were treated with siRNA targeting cGAS or negative control, stimulated with poly(dA:dT), and analyzed by WB with the indicated primary antibodies. (*E*) A549-HARQ-2B3 cells were transfected with cGAS and analyzed by WB with the indicated antibodies. (*F*) 293T/17 cells were cotransfected with cGAS (0.5 μg), STING (HARQ haplotype, 0.4 μg), IRF3 (0.3 μg), Vip-Luc (0.2 μg), and internal control pRL-TK (0.1 μg) for 24 h as indicated. Cell lysates were analyzed by dual-luciferase assay. GS/AA, a catalytic dead cGAS mutant. Vip-Luc, a *viperin*-promoter-driven Firefly luciferase reporter. Data are mean ± SD (*n* = 3 per group) and were compared by two-tailed Student *t* test. (*G*) 293T/17 cells were transfected with each cGAS for 24 h. 2′3′-cGAMP in the cell lysates and culture supernatants (sup.) were analyzed by ELISA. Data are mean ± SD (*n* = 3 per group) and were compared by two-tailed Student *t* test. (*H* and *I*) 293T/17 cells were transfected with cGAS for 18 h, then cocultured with A549-HARQ-2B3 cells (*H*) for another 5 h. The 2′3′-cGAMP in the culture media was measured by mass spectrometry (also refer to *SI Appendix*, Fig. S6). Data are mean ± SD (*n* = 3 per group). U.D., under detection limit. The coculture cell lysates were analyzed by WB (*I*). Black arrow, cGAS-V5; gray arrow, full-length STING-V5; white arrow, cleaved STING-V5. Quantification of the STING cleavage levels was in a bar graph. The full-length (FL; gray bar) and cleaved (white bar) STING-V5 signal were divided by the total STING-V5 signal of each lane for the relative ratio in percentage. Data are mean ± SD (*n* = 3 per group).

To understand whether DNA stimuli emerged in cytoplasm upon DENV infection, the subcellular localization of endogenous DNA in DENV-infected cells was examined by IFA with anti-DNA antibody (Fig. 2*A*). As expected, the nuclear DNA signal colocalized with DAPI counterstaining, whereas the cytoplasmic DNA signal seemed mostly embedded in mitochondria (Fig. 2*A*). Some DNA signals, neither concealed in the nucleus nor mitochondria, were detected in the cytosol of DENV-infected cells (Fig. 2*A*, white arrows). Because mitochondrial DNA (mtDNA) can be released from the mitochondria to trigger innate immune responses (21), we established mtDNA-depleted A549-HARQ ρ0 cells (Fig. 2*B*) to address the possible role of mtDNA in the STING cleavage event. In the presence or absence of mtDNA, the difference in DENV-induced cell death (*SI Appendix*, Fig. S5) and STING cleavage were marginal (Fig. 2*C*), which suggests that mtDNA is not the intrinsic stimuli enhancing STING cleavage.

Because all tested DNA preparations enhanced the HARQ haplotype cleavage by DENV protease (*SI Appendix*, Fig. S4*C*), we checked whether the DNA sensing pathway per se participates in this cleavage phenomenon. In A549-HARQ-2B3 cells, DNA-enhanced STING cleavage was attenuated by siRNA targeting cGAS (Fig. 2*D*), whereas overexpression of human cGAS facilitated the cleavage event (Fig. 2*E*). We further used the cGAS catalytic-dead mutant (GS/AA) (16) to understand whether the cGAS enzyme activity is involved. The synthase activity of cGAS was required to potentiate STING signaling of HARQ (Fig. 2*F*). Furthermore, the cGAS product 2′3′-cGAMP was detected in lysates and culture supernatants of cells transfected with WT cGAS but not much with the catalytic-dead mutant GS/AA (Fig. 2*G*).

Because cGAS synthesizes 2′3′-cGAMP as a second messenger to spread intrinsic immunity to bystander cells (14), we addressed whether this STING cleavage could be enhanced by adjacent cells expressing cGAS. 2′3′-cGAMP-producing 293T/17 cells were prepared by transfection of plasmid DNA encoding human cGAS, then cocultured with A549-HARQ-2B3 cells (Fig. 2*H* and *SI Appendix*, Fig. S6). The sensitive STING HARQ in A549 cells was readily cleaved when cocultured with 293T/17 cells transfected with WT but not GS/AA-mutated cGAS (Fig. 2*I*). These data suggest that cGAS participates in regulating the DENV protease-mediated cleavage by producing 2′3′-cGAMP.

### 2′3′-cGAMP Is Responsible for the DNA-Enhanced STING Cleavage of HARQ Haplotype.

**Fig. 3.**
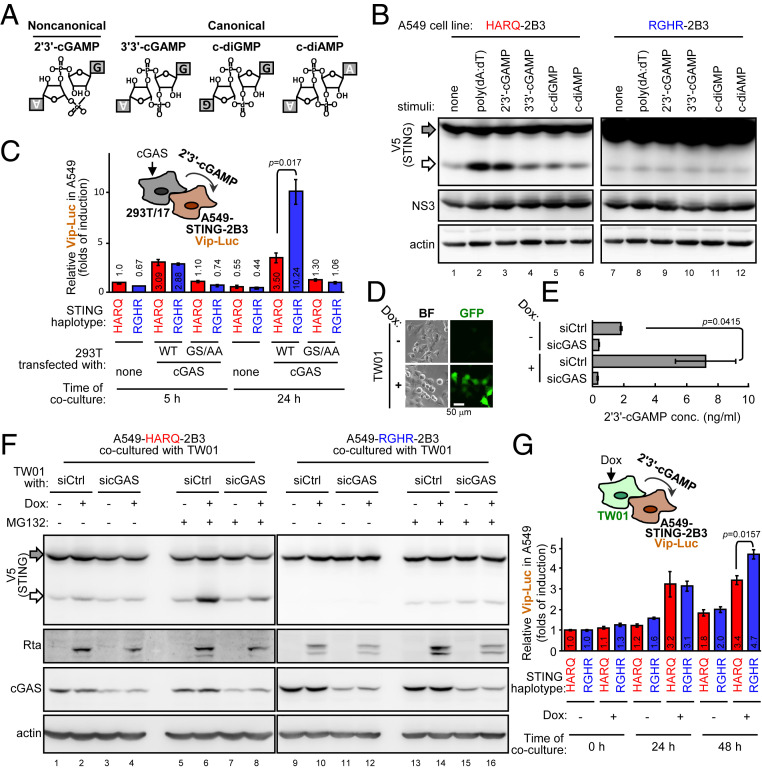
2′3′-cGAMP enhances DENV protease-mediated cleavage of STING HARQ haplotype. (*A* and *B*) The A549-HARQ-2B3 or A549-RGHR-2B3 cells were stimulated with the indicated CDNs (10 μg/mL) (*A*) or poly(dA:dT) (1 μg/mL) for 4.5 h and then analyzed by WB (*B*). (*C*) A549-HARQ-2B3 or A549-RGHR-2B3 cells were further stably transduced with the lentiviral vector harboring Vip-Luc and then used in the coculture experiment as described in Fig. 2*H*. The luciferase activity in the coculture cell lysates was analyzed. Data are mean ± SD (*n* = 3 per group) and were compared by two-tailed Student *t* test. (*D*) TW01 cells harboring the doxycycline (Dox)-inducible EBV reactivation system were cultured with (+) or without (−) Dox (0.1 μg/mL, 24 h). BF, bright field. (*E* and *F*) TW01 cells were transfected with siRNA targeting negative control (siCtrl) or cGAS (sicGAS) for 54 h, then cocultured with A549-STING-2B3 cells with (+) or without (−) Dox (0.1 μg/mL, 24 h). The proteasome inhibitor MG132 (1 μM) treatment was added 6 h before harvest. 2′3′-cGAMP in the TW01 cell lysates (*E*) without coculture were analyzed by ELISA. Data are mean ± SD (*n* = 3 per group) and were compared by two-tailed Student *t* test. The cocultured cell lysates were analyzed by WB (*F*). Gray arrow, full-length STING-V5; white arrow, cleaved STING-V5. (*G*) A549-HARQ-2B3-Vip-Luc or A549-RGHR-2B3-Vip-Luc cells were cocultured with TW01 cells with (+) or without (−) Dox (0.25 μg/mL). The luciferase activity in the coculture cell lysates was analyzed. Data are mean ± SD (*n* = 3 per group) and were compared by two-tailed Student *t* test.

To understand whether 2′3′-cGAMP is a specific CDN directing the cleavage event, several 2′3′-cGAMP–like CDNs (Fig. 3*A*) were delivered to A549 cells stably coexpressing DENV protease and each STING. Enhanced STING cleavage was readily observed in cells harboring the HARQ haplotype treated with poly(dA:dT) and 2′3′-cGAMP but not other CDNs (Fig. 3*B*, lanes 1–6). None of the CDNs tested could enhance the cleavage of the RGHR haplotype (Fig. 3*B*, lanes 7–12). Consequently, coculture of cGAS-expressing 293T/17 cells (Fig. 3*C*) elicited greater STING signaling in A549-RGHR-2B3 than A549-HARQ-2B3 cells (Fig. 3*C*). Thus, 2′3′-cGAMP accounts explicitly for the DNA-enhanced, DENV protease-mediated cleavage of the STING HARQ haplotype.

Because mtDNA seemed irrelevant to STING cleavage (Fig. 2*C*), we next sought to examine whether other exogenous DNA could serve as the cytoplasmic DNA stimulator triggering 2′3′-cGAMP production. We chose the doxycycline (Dox)-inducible Epstein–Barr virus (EBV) reactivation cell line TW01 (22) as a controlled DNA pathogen model for the coculture experiment because EBV is a common human DNA pathogen harboring latency and reactivation characteristics. In the presence of Dox, TW01 cells reactivated the recombinant EBV-expressing GFP (Fig. 3*D*) and produced 2′3′-cGAMP (Fig. 3*E*) to activate STING signaling (*SI Appendix*, Fig. S7). We then tested whether this EBV reactivation model could trigger STING cleavage in the coculture system. Upon EBV reactivation, the enhancement of HARQ cleavage appeared to be marginal (Fig. 3*F*, lanes 1–4) but became apparent in the presence of the proteasome inhibitor MG132 (Fig. 3*F*, lanes 5–8), which has been shown to enhance the cleaved product signal of STING (8). Consistently, RGHR cleavage was not affected by EBV reactivation (Fig. 3*F*, lanes 9–16). Furthermore, neighboring EBV reactivation-induced STING signaling was greater in A549-RGHR-2B3 than A549-HARQ-2B3 cells after 48 h (Fig. 3*G*), which suggests that a bystander DNA pathogen could manipulate DENV protease-regulated STING signaling, and this phenomenon was STING haplotype-specific (*SI Appendix*, Fig. S8).

### The Resistant RGHR Induces Stronger Innate Immunity than the Sensitive HARQ in Response to DENV Infection.

**Fig. 4.**
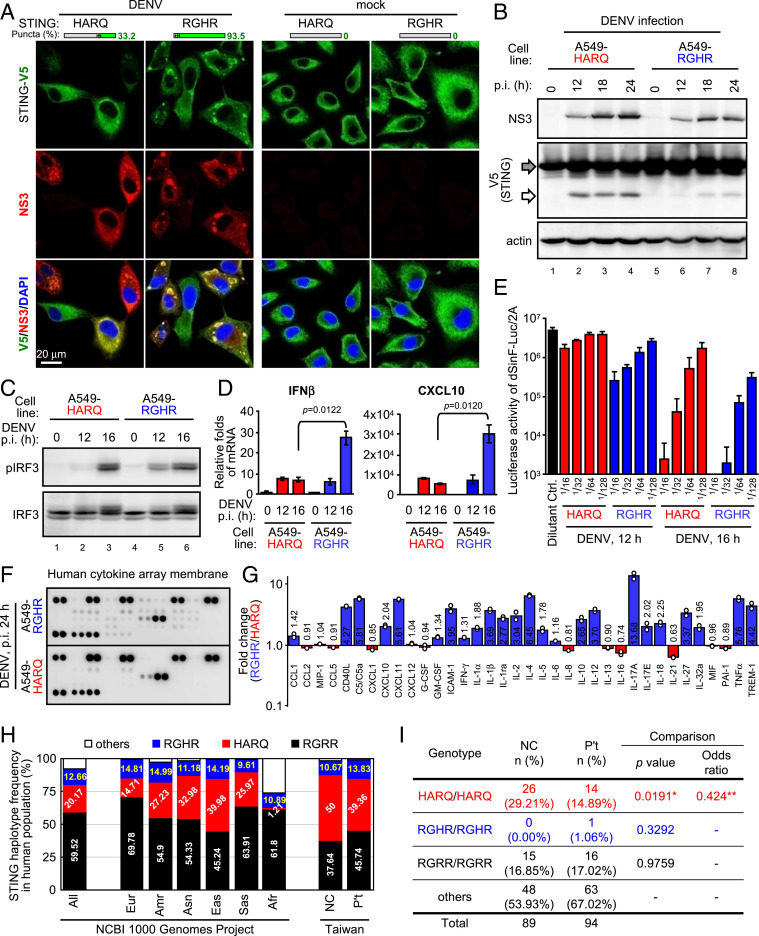
DENV protease specifically restricts host innate immunity in cells harboring STING haplotype HARQ. (*A*–*G*) A549-HARQ and A549-RGHR cells were infected with DENV (moi 5–10) for the indicated time, then analyzed by IFA (*A*; by Olympus FV1000, 1,024 × 1,024 pixels, gain = 1–3×; the bars showed the percentage of cells harboring STING puncta by mean ± SD, *n* = 3 per group), WB (*B* and *C*), and RT-qPCR (*D*; data are mean ± SD, *n* = 3 per group, and were compared by two-tailed Student *t* test). The culture supernatants were collected (*E*–*G*), UV-irradiated, diluted with fresh culture media, then used to treat Vero for 20 h as indicated. The conditioned Vero cells were infected with IFN-sensitive dSinF-Luc/2A sindbis reporter virus harboring firefly luciferase for 24 h and then harvested for luciferase activity (*E*; Dilutant Ctrl, fresh complete culture media; *n* = 3, biological replicates). The culture supernatants were reacted with human cytokine array membranes as indicated (*F*; the complete image data can be found in *SI Appendix*, Fig. S12), then quantified as fold change of RGHR/HARQ (*G***;** shown as average, *n* = 2 technical replicates; blue and red, cytokines up-regulated in A549-RGHR and -HARQ respectively). (*H*) The distribution of STING haplotypes in subhuman population. The haplotype information was analyzed by using the web-based application LDlink with the NCBI 1000 Genomes Project data as indicated. Afr, African; Amr, Ad Mix American; Asn, Asian; Eas, East Asian; Eur, European; Sas, South Asian. For the Taiwan population, genomic DNA from normal control Taiwanese (23) (NC, *n* = 89) and Taiwanese people with DENV infection (P’t, *n* = 94) were analyzed for STING haplotypes (the representative STING haplotype genotyping data are in *SI Appendix*, Fig. S13). (*I*) Genomic DNA derived from residual specimen of people with DENV infection (P’t) in Taiwan and from normal control Taiwanese was analyzed for STING haplotypes. Shows the number (n) and frequency (%) of each STING genotype in NC and P’t groups (the detailed information is in *SI Appendix*, Fig. S14). Each STING genotype in NC and P’t was compared with all others by 2 × 2 contingency tables and compared by χ^2^ test. Total samples were 94 NC and 102 P’t. The missing values in SNP identification resulted in the genotyping data exclusion of 5 NC and 8 P’t samples. *The *P* value is significant at 0.019142. **95% CI of the odds ratio: 0.2046–0.8789; Significance level: *P =* 0.021.

Next, we tested whether the vulnerability of STING to DENV protease is reflected in DENV infection. The ectopic RGHR formed more STING activation aggregates than did HARQ in DENV-infected A549 cells (Fig. 4*A*) because the RGHR remained more resistant than HARQ to DENV protease after DENV infection (Fig. 4*B*, white arrow). In contrast to the sensitive HARQ, the resistant RGHR induced a higher level of antiviral IFNβ induction and signaling (Fig. 4 *C* and *D* and *SI Appendix*, Figs. S9 and S10) that results in a stronger antiviral activity (Fig. 4*E*) against DENV (*SI Appendix*, Fig. S11). More than IFNβ, the human cytokine array revealed that RGHR caused a higher level of cytokine production profile than did HARQ (Fig. 4 *F* and *G* and *SI Appendix*, Fig. S12) in response to DENV infection.

Both the higher viral burden and induction of a robust host immune response contribute to dengue pathogenesis (24). We asked whether human STING haplotypes are related to DENV pathogenesis in a Taiwan population. STING haplotype frequency varies among different subhuman populations (Fig. 4*H*). Although the HARQ frequency is 20.17% in the global population, it is 50% in a normal Taiwan population (23) and 39.36% in Taiwanese people with DENV infection (Fig. 4*H* and *SI Appendix*, Fig. S13). By comparing HARQ/HARQ versus the other genotypes, we found that the HARQ homozygote frequency was significantly reduced (χ^2^ test, *P* = 0.0191) in Taiwanese people with DENV infection (Fig. 4*I* and *SI Appendix*, Fig. S14). Accordingly, the HARQ/HARQ STING genotype may be a protective factor in DENV-infected Taiwanese people (odds ratio = 0.424, 95% CI: 0.2046–0.8789) (Fig. 4*I*), protecting host against excessive STING-mediated responses. Side effects of host innate immune responses will play a more prominent role than the viral load in contributing to DENV pathogenesis if the frequency of the HARQ/HARQ genotype is globally low in DENV-infected people. Nevertheless, here we demonstrated that the DENV protease sensitivity of STING is the cause rather than the consequence, governing cytokine production and virus replication upon DENV infection.

## Discussion

Many viruses evolve strategies to down-regulate host innate immunity-related factors for a better replication environment. STING is conserved in many species (25), mediating antiviral responses against infection. A conserved STING mutation dampening IFN activation contributes to herpes simplex virus replication in bats (26). DENV cleaves human STING to antagonize the host antiviral system. A DENV protease-resistant STING found in murine or nonhuman primates (6–8) suggested that STING might be a host factor restricting DENV replication among different species. Natural selection might result in the fittest STING haplotype in the subhuman population geographically in response to deadly infectious diseases, including but not limited to dengue. Despite many gene variants associated with dengue illness (27), the iceberg theory of disease severity in humans after DENV infection (28) suggests that the pathogenic factors of DENV do not act alone. Our coculture cell model showed evidence of a human genetic factor together with a bystander coinfection possibly contributing to DENV pathogenesis. The DENV protease-regulated human STING antiviral signaling is haplotype-specific, and the cleavage of protease-sensitive HARQ can be further manipulated by 2′3′-cGAMP derived from a neighboring cell with DNA pathogen infection.

The linkage between mitochondria and innate immunity has been intensively studied in recent decades. The virus-induced dislocation of cellular components would affect the fate of the host after infection. The release of cytochrome *c* from mitochondria assembles the intrinsic apoptosome (29), thereby leading to the death of host cells in response to virus infection. The mtDNA concealed in mitochondria could be released to activate the inflammasome and increase levels of inflammatory cytokines as a consequence (21). The release of mtDNA should theoretically activate the cGAS-STING pathway in cytoplasm. However, the induction of antiviral IFN by the passive release of mtDNA along with apoptosis at a relatively late stage of virus infection might not occur unless the release is actively induced earlier in infection.

Depletion of mtDNA in human cells has been used in various metabolism studies but rarely in innate immunity. Despite possible alterations in metabolic preferences, we clearly showed that mtDNA is not required for enhancing STING cleavage upon DENV infection. If the DNA-enhanced STING cleavage solely depends on 2′3′-cGAMP, extrinsic 2′3′-cGAMP derived from bystander cells or microorganisms, rather than intrinsic 2′3′-cGAMP synthesized from cGAS-sensing endogenous mtDNA, could be the missing pathogenic factor of DENV. The neutrophil extracellular traps (NETs) (30) were composed of neutrophil granular proteins and DNA that form extracellular fibers binding to pathogens. Thus, the NETs might be considered with any involvement of cellular DNA physiologically or pathologically.

STING is an endoplasmic reticulum adaptor protein awaiting its agonists to activate innate immunity. Binding of 2′3′-cGAMP could trigger STING conformational changes resulting in high-order oligomerization (31, 32) and translocation to the Golgi apparatus (33). The conformational changes of STING might make particular STING structurally accessible to DENV protease or lead STING moving to a certain subcellular compartment where it meets DENV protease and then be cleaved. Extensive studies understanding the structure, subcellular localization, and interaction between DENV protease and different STINGs in the presence or absence of 2′3′-cGAMP would further clarify these hypotheses.

An early-onset autoimmune disorder, Aicardi–Goutières syndrome, has been linked to chronic activation of the cGAS-STING pathway invoking superfluous innate immune responses (34). DENV-induced illness might result from hyperactive interferonopathy (35) or dysregulated STING-induced vasculopathy (36). Our findings reveal a previously neglected mechanism of how neighboring cells modulate DENV protease to antagonize innate immunity in a human STING haplotype-specific manner, potentially renewing DENV pathogenesis and affecting DENV disease prognosis. Global comprehensive studies monitoring STING haplotypes and coinfection pathogens in DENV patients seem warranted to provide the missing link and important clues of DENV pathogenesis.

## Materials and Methods

### Plasmids.

We used the previously described plasmids expressing HA-mSTING-V5 (7), HA-MFN2-V5 (19), NS2B3 (WT or S135A)-Flag (37), and Vip-Luc (38). Plasmids expressing STING haplotypes (*SI Appendix*, Fig. S1) were obtained by single-primer mutagenesis (39) with the human HARQ haplotype template (7) and multiple primers (*SI Appendix*, Table S1). The lentiviral vector pLKO.1_AS3w.puro was used for cDNA expression and was obtained from the National RNAi Core Facility (Academia Sinica, Taiwan); the pTY lentiviral expression system was from Lung-Ji Chang (40). The cDNA of STING haplotypes was subcloned in the pLKO.1_AS3w.puro vector with primers Asc1-HA and BGH pA/R; the Vip-Luc was subcloned in the pTY vector with EcoRI-Luc(1653-1632) and EcoRI-FLuc (1–16) primers. The human cGAS cDNA was amplified from total cDNA from A549 cells by PCR with hcGAS primers and cloned in the shuttle vector pJET1.2 (K1231, Thermo). The cDNA of human cGAS was then subcloned in the N-terminal HA-tagged, C-terminal V5-tagged expression vector with Xho1-hcGAS (4–20) and hcGAS-Sac2(1566-1540) primers. The GS/AA catalytic dead cGAS construct was generated by single-primer mutagenesis with the primer cGAS(GS/AA). The plasmid constructs were checked by restriction enzymes and validated by DNA sequencing. Information about the primers is in *SI Appendix*, Table S1.

### Viruses and Cell Lines.

Propagation and titration of DENV-2 PL046 were as described (41). The IFN-sensitive dSinF-Luc/2A sindbis reporter virus (42) was amplified and titrated as described (7). Sources of cell lines used in this study were summarized in *SI Appendix*, Table S2. Human lung epithelial carcinoma A549 cells were cultured in F-12K medium containing 10% fetal bovine serum (FBS). Human embryonic kidney *293T/17* cells were grown in DMEM containing 10% FBS. African green-monkey kidney Vero cells were cultured in MEM containing 10% FBS. TW01 cells with EBV latent infection (22) were cultured in RPMI medium 1640 containing 10% FBS. The EBV reactivation was triggered by EBV Rta protein in a doxycycline-inducible expression system. All cells were PCR-confirmed as mycoplasma-negative. To establish A549 cells stably coexpressing DENV protease and each STING, A549-NS2B3(WT) or A549-NS2B3(S135A) cells (7) were transduced with the lentiviral vector harboring HA-STING(HARQ)-V5 or HA-STING(RGHR)-V5, then selected with 10 μg/mL puromycin. The ρ0 cells were established by culturing cells in a low dose of ethidium bromide (EtBr)-containing medium as described (43). Briefly, the cells were cultured in Complete media containing 50 ng/mL EtBr, 50 μg/mL uridine, and 100 μg/mL sodium pyruvate (44) for more than 6 mo. The depletion of mtDNA was revealed by PCR and PicoGreen live DNA staining. For cells stably containing Vip-Luc, a Firefly luciferase reporter driven by the *viperin* promoter (38), cells were transduced with the pTY lentiviral vector harboring Vip-Luc.

### Western Blot Analysis.

Cells were lysed with RIPA buffer (10 mM Tris, pH 7.5, 5 mM EDTA, 150 mM NaCl, 0.1% SDS, 1% Triton X-100, 1% sodium deoxycholate) containing a mixture of protease inhibitors and phosphatase inhibitors. Equivalent amounts of proteins determined by the DC Protein Assay Kit were separated on SDS/PAGE and transferred to a nitrocellulose membrane (XR-IGE-10600003, Amersham). Nonspecific antibody binding sites were blocked with skim milk in phosphate-buffered saline (PBS) with 0.1% Tween 20 (PBST), then reacted with the indicated primary antibodies and incubated with appropriate horseradish peroxidase-conjugated secondary antibodies. Signals were detected by Chemiluminescence HRP Substrate (Millipore). Information for antibodies are in *SI Appendix*, Table S3. The Proteome Profiler Human Cytokine Array Kit (ARY005B, R&D Systems) membrane spotted in duplicate with 36 different cytokine antibodies was used for the cytokine array analysis. Images were quantified by using ImageJ. Full-length blot images are in *SI Appendix*, Fig. S12.

### Immunofluorescence Assay.

Cells were fixed with 4% paraformaldehyde in PBS for 30 min at room temperature, then permeabilized in PBS containing 0.5% Triton X-100 for 10 min. After blocking with skim milk in PBS for 30 min, cells were incubated with primary antibodies diluted in skim milk in PBS overnight, then with appropriate secondary antibodies for 1 h at room temperature followed by nuclear DAPI counterstaining (0.25 ng/mL, 7 min). Cells were photographed under a confocal fluorescence microscope (FV1000, Olympus) with a 100× objective. For MitoTracker staining, cells were stained with MitroTracker Red (0.1 ng/mL) for 10–15 min before 15-min fixation with prewarmed 4% paraformaldehyde in PBS. The PicoGreen live staining followed the manufacturer’s instructions (Thermo Fisher Scientific) and was observed by fluorescence microscopy. Information on antibodies is in *SI Appendix*, Table S3.

### Immunoprecipitation–Western Blot Analysis.

Cells were lysed with RIPA buffer containing a mixture of protease inhibitors. Cell lysates were incubated with magnetic beads at 4 °C for 30 min. The precleared samples were then immunoprecipitated with the indicated antibodies overnight at 4 °C. Next, 4% bovine serum albumin (BSA) preblocked magnetic beads were added into the lysate-antibody mixture at 4 °C for 1 h. The immune complex was then washed with RIPA three times at 4 °C for 10 min. Proteins were eluted with 2× sample buffer and examined by Western blot analysis with the indicated antibodies.

### Reporter Assay.

Cell lysates were harvested and quantified by using a Dual-Luciferase Assay System (Promega). Relative luciferase activities were normalized with the internal control *renilla* luciferase derived from pRL-TK. Luciferase activity of A549-STING-2B3-Vip-Luc cocultured with 293T/17 or TW01 were also analyzed by using the Dual-Luciferase Assay System with the GLOMAX Multi+ Microplate Multimode Reader (Promega).

### Preparation and Treatment of Nucleic Acids and CDNs.

The synthetic B form DNA analog poly(dA:dT) and CDNs were from InvivoGen. The plasmid DNA was prepared by using a QIAprep Spin Miniprep kit (Qiagen). The bacterial DNA derived from ECOS 101 Competent Cells (Yeastern Biotech) was prepared by using the Presto Mini gDNA Bacteria Kit (Geneaid Biotech). Total DNA and w/o mtDNA were total DNA derived from A549 and A549-ρ0 cells, respectively, by using the PureLink Genomic DNA Mini Kit (Thermo Fisher Scientific). The small interfering RNAs (siRNA) were from Ambion or Invitrogen. Both DNA and siRNAs were delivered by using Lipofectamine 2000 transfection reagent (Thermo Fisher Scientific). For siRNA transfection, cells were sequentially transfected with 50 nM siRNAs for 48 h, followed by another 24 h of siRNA transfection. For delivery of CDNs, cells were incubated with the indicated CDN mixed with permeabilization buffer (50 mM Hepes, pH 7.3, 100 mM KCl, 3 mM MgCl_2_, 85 mM sucrose, 0.2% BSA, 0.1 mM dithiothreitol [DTT], 1 mM adenosine triphosphate [ATP], 0.1 mM guanosine triphosphate [GTP]) and digitonin (2.5 μg/mL) at 37 °C for 30 min. Cells were washed twice with PBS, then the mixture was removed and fresh culture medium was added and incubated for 4 h. A detailed list of concentrations for all of the nucleic acids used for experiments can be found in *SI Appendix*, Table S4.

### 2′3′-cGAMP Measurement.

For measuring 2′3′-cGAMP, an ELISA kit based on the competition between 2′3′-cGAMP and a 2′3′-cGAMP-HRP conjugate (Item No. 501700, Cayman Chemical) was used and described in the figure legends. For the mass spectrometry-based quantification of 2′3′-cGAMP, the samples of culture medium were analyzed by ultraperformance liquid chromatography (UPLC)-positive electrospray ionization-mass/mass spectrometry (MS/MS). The standard of 2′3′-cGAMP was used for constructing daughter ion scan spectra for identity confirmation and also for quantitative calculation by multiple reaction monitoring. Separation was carried out by reverse-phase UPLC on an Acquity UPLC BEH C18 1.7 μm column, 2.1 mm × 50 mm (Waters) at 30 °C. The elution started from 99% mobile phase A (0.1% formic acid in ultrapure water) and 1% mobile phase B (0.1% formic acid in methanol), held at 1% B for 0.5 min, raised to 95% B in 1.5 min, held at 95% B for 1.5 min, and then lowered to 1% B in 0.5 min. The column was equilibrated by pumping 1% B for 3.5 min. The flow rate was set at 0.2 mL/min with injection volume of 5 μL for standard and 7.5 μL for samples. Mass spectra and chromatogram were acquired in negative electrospray ionization mode (ES-) using Waters TQ-XS LC mass spectrometer. The identity and contents of 2′3′-cGAMP were determined by LC retention time plus MS/MS spectra and quantified with the LC peak areas based on the ion mass transition.

### RT-qPCR.

Total RNA was isolated by an RNeasy Mini Kit (Qiagen), and the cDNA was reverse transcribed by using a High Capacity cDNA Reverse Transcription Kit (Applied Biosystems). qPCR was then carried out with the Fast SYBR Green Master Mix (Applied Biosystems) by using the specific primers listed in *SI Appendix*, Table S1. The relative expression of IFNβ and CXCL10 were normalized to that of actin.

### Genotyping of STING Haplotype.

The genomic DNA was prepared by using the PureLink Genomic DNA Mini Kit. The two SNPs rs11554776 (amino acid [a.a.] 71) and rs7380824 (a.a. 293) for human STING were identified by TaqMan SNP Genotyping Assays with TaqMan Genotyping Master Mix. For the SNPs, rs78233829 (a.a. 230) and rs1131769 (a.a. 232), the genome was partially amplified by PCR (with primers TMEM173-F and TMEM173-R) and identified by DNA sequencing. Information about the primers is in *SI Appendix*, Table S1. Information about the samples from Taiwan BioBank can be found elsewhere (23); the collection and analysis of samples from Taiwanese people with DENV infection followed the related protocols approved by The Institutional Review Board (IRB) of National Cheng Kung University Hospital (IRB B-ER-106-150). All participants gave their informed consent or assent for inclusion before they participated in the study. Genotyping data were excluded if SNP missing values failed in STING haplotype identification.

### Quantification and Statistical Analysis.

Data are shown as mean ± SD. Two-tailed Student *t* test was used for comparing two groups as described in figure legends. Two-sided χ^2^ test with 2 × 2 contingency table was used for comparing the STING genotype in NC and P’t groups as described in figure legends. *P* < 0.05 was considered statistically significant.

### Data Availability.

The web-based application LDlink (https://ldlink.nci.nih.gov) using the National Center for Biotechnology Information (NCBI) 1000 Genomes Project data are available from the National Cancer Institute, NIH. All data supporting the findings of the study are included in the paper and *SI Appendix*.

## Supplementary Material

Supplementary File

## References

[1] J. C. Lee.; UK IBD Genetics Consortium, Human SNP links differential outcomes in inflammatory and infectious disease to a FOXO3-regulated pathway. Cell 155, 57–69 (2013). 24035192 10.1016/j.cell.2013.08.034 PMC3790457

[2] D. Ge., Genetic variation in IL28B predicts hepatitis C treatment-induced viral clearance. Nature 461, 399–401 (2009). 19684573 10.1038/nature08309

[3] A. Sakuntabhai., A variant in the CD209 promoter is associated with severity of dengue disease. Nat. Genet. 37, 507–513 (2005). 15838506 10.1038/ng1550 PMC7096904

[4] K. Djamiatun., Toll-like receptor 4 polymorphisms in dengue virus-infected children. Am. J. Trop. Med. Hyg. 85, 352–354 (2011). 21813858 10.4269/ajtmh.2011.10-0728 PMC3144836

[5] C. Cruz-Oliveira., Receptors and routes of dengue virus entry into the host cells. FEMS Microbiol. Rev. 39, 155–170 (2015). 25725010 10.1093/femsre/fuu004

[6] S. Aguirre., DENV inhibits type I IFN production in infected cells by cleaving human STING. PLoS Pathog. 8, e1002934 (2012). 23055924 10.1371/journal.ppat.1002934 PMC3464218

[7] C. Y. Yu., Dengue virus targets the adaptor protein MITA to subvert host innate immunity. PLoS Pathog. 8, e1002780 (2012). 22761576 10.1371/journal.ppat.1002780 PMC3386177

[8] A. C. Stabell., Dengue viruses cleave STING in humans but not in nonhuman primates, their presumed natural reservoir. eLife 7, e31919 (2018). 29557779 10.7554/eLife.31919 PMC5860865

[9] Y. T. Kao, M. M. C. Lai, C. Y. Yu, How dengue virus circumvents innate immunity. Front. Immunol. 9, 2860 (2018). 30564245 10.3389/fimmu.2018.02860 PMC6288372

[10] S. Aguirre., Dengue virus NS2B protein targets cGAS for degradation and prevents mitochondrial DNA sensing during infection. Nat. Microbiol. 2, 17037 (2017). 28346446 10.1038/nmicrobiol.2017.37 PMC7457382

[11] H. Liu., Endoplasmic reticulum protein SCAP inhibits dengue virus NS2B3 protease by suppressing its K27-linked polyubiquitylation. J. Virol. 91, e02234-16 (2017). 28228593 10.1128/JVI.02234-16 PMC5391462

[12] L. Sun, J. Wu, F. Du, X. Chen, Z. J. Chen, Cyclic GMP-AMP synthase is a cytosolic DNA sensor that activates the type I interferon pathway. Science 339, 786–791 (2013). 23258413 10.1126/science.1232458 PMC3863629

[13] E. J. Diner., The innate immune DNA sensor cGAS produces a noncanonical cyclic dinucleotide that activates human STING. Cell Rep. 3, 1355–1361 (2013). 23707065 10.1016/j.celrep.2013.05.009 PMC3706192

[14] A. Ablasser., Cell intrinsic immunity spreads to bystander cells via the intercellular transfer of cGAMP. Nature 503, 530–534 (2013). 24077100 10.1038/nature12640 PMC4142317

[15] J. Wu., Cyclic GMP-AMP is an endogenous second messenger in innate immune signaling by cytosolic DNA. Science 339, 826–830 (2013). 23258412 10.1126/science.1229963 PMC3855410

[16] F. Civril., Structural mechanism of cytosolic DNA sensing by cGAS. Nature 498, 332–337 (2013). 23722159 10.1038/nature12305 PMC3768140

[17] C. Chiang, M. U. Gack, Post-translational control of intracellular pathogen sensing pathways. Trends Immunol. 38, 39–52 (2017). 27863906 10.1016/j.it.2016.10.008 PMC5580928

[18] G. Yi., Single nucleotide polymorphisms of human STING can affect innate immune response to cyclic dinucleotides. PLoS One 8, e77846 (2013). 24204993 10.1371/journal.pone.0077846 PMC3804601

[19] C. Y. Yu., Dengue virus impairs mitochondrial fusion by cleaving mitofusins. PLoS Pathog. 11, e1005350 (2015). 26717518 10.1371/journal.ppat.1005350 PMC4696832

[20] T. Saitoh., Atg9a controls dsDNA-driven dynamic translocation of STING and the innate immune response. Proc. Natl. Acad. Sci. U.S.A. 106, 20842–20846 (2009). 19926846 10.1073/pnas.0911267106 PMC2791563

[21] K. Shimada., Oxidized mitochondrial DNA activates the NLRP3 inflammasome during apoptosis. Immunity 36, 401–414 (2012). 22342844 10.1016/j.immuni.2012.01.009 PMC3312986

[22] Y. Y. Lan., Epstein-Barr virus Zta upregulates matrix metalloproteinases 3 and 9 that synergistically promote cell invasion in vitro. PLoS One 8, e56121 (2013). 23409137 10.1371/journal.pone.0056121 PMC3567054

[23] W. H. Pan., Han Chinese cell and genome bank in Taiwan: Purpose, design and ethical considerations. Hum. Hered. 61, 27–30 (2006). 16534213 10.1159/000091834

[24] World Health Organization, Dengue Guidelines for Diagnosis, Treatment, Prevention and Control, (WHO/TDR, Geneva, 2009). 23762963

[25] X. Wu., Molecular evolutionary and structural analysis of the cytosolic DNA sensor cGAS and STING. Nucleic Acids Res. 42, 8243–8257 (2014). 24981511 10.1093/nar/gku569 PMC4117786

[26] J. Xie., Dampened STING-dependent interferon activation in bats. Cell Host Microbe 23, 297–301.e4 (2018). 29478775 10.1016/j.chom.2018.01.006 PMC7104992

[27] M. G. Guzman, D. J. Gubler, A. Izquierdo, E. Martinez, S. B. Halstead, Dengue infection. Nat. Rev. Dis. Primers 2, 16055 (2016). 27534439 10.1038/nrdp.2016.55

[28] J. L. Kyle, E. Harris, Global spread and persistence of dengue. Annu. Rev. Microbiol. 62, 71–92 (2008). 18429680 10.1146/annurev.micro.62.081307.163005

[29] Z. T. Schafer, S. Kornbluth, The apoptosome: Physiological, developmental, and pathological modes of regulation. Dev. Cell 10, 549–561 (2006). 16678772 10.1016/j.devcel.2006.04.008

[30] G. Sollberger, D. O. Tilley, A. Zychlinsky, Neutrophil extracellular traps: The biology of chromatin externalization. Dev. Cell 44, 542–553 (2018). 29533770 10.1016/j.devcel.2018.01.019

[31] G. Shang, C. Zhang, Z. J. Chen, X. C. Bai, X. Zhang, Cryo-EM structures of STING reveal its mechanism of activation by cyclic GMP-AMP. Nature 567, 389–393 (2019). 30842659 10.1038/s41586-019-0998-5 PMC6859894

[32] C. Zhang., Structural basis of STING binding with and phosphorylation by TBK1. Nature 567, 394–398 (2019). 30842653 10.1038/s41586-019-1000-2 PMC6862768

[33] X. Gui., Autophagy induction via STING trafficking is a primordial function of the cGAS pathway. Nature 567, 262–266 (2019). 30842662 10.1038/s41586-019-1006-9 PMC9417302

[34] T. Li, Z. J. Chen, The cGAS-cGAMP-STING pathway connects DNA damage to inflammation, senescence, and cancer. J. Exp. Med. 215, 1287–1299 (2018). 29622565 10.1084/jem.20180139 PMC5940270

[35] M. P. Rodero, Y. J. Crow, Type I interferon-mediated monogenic autoinflammation: The type I interferonopathies, a conceptual overview. J. Exp. Med. 213, 2527–2538 (2016). 27821552 10.1084/jem.20161596 PMC5110029

[36] J. D. Warner., STING-associated vasculopathy develops independently of IRF3 in mice. J. Exp. Med. 214, 3279–3292 (2017). 28951494 10.1084/jem.20171351 PMC5679177

[37] C. Y. Yu, Y. W. Hsu, C. L. Liao, Y. L. Lin, Flavivirus infection activates the XBP1 pathway of the unfolded protein response to cope with endoplasmic reticulum stress. J. Virol. 80, 11868–11880 (2006). 16987981 10.1128/JVI.00879-06 PMC1642612

[38] Y. L. Chan, T. H. Chang, C. L. Liao, Y. L. Lin, The cellular antiviral protein viperin is attenuated by proteasome-mediated protein degradation in Japanese encephalitis virus-infected cells. J. Virol. 82, 10455–10464 (2008). 18768981 10.1128/JVI.00438-08 PMC2573197

[39] O. Makarova, E. Kamberov, B. Margolis, Generation of deletion and point mutations with one primer in a single cloning step. Biotechniques 29, 970–972 (2000). 11084856 10.2144/00295bm08

[40] L. J. Chang, V. Urlacher, T. Iwakuma, Y. Cui, J. Zucali, Efficacy and safety analyses of a recombinant human immunodeficiency virus type 1 derived vector system. Gene Ther. 6, 715–728 (1999). 10505094 10.1038/sj.gt.3300895

[41] Y. L. Lin., Study of Dengue virus infection in SCID mice engrafted with human K562 cells. J. Virol. 72, 9729–9737 (1998). 9811707 10.1128/jvi.72.12.9729-9737.1998 PMC110483

[42] P. Y. Huang, J. H. Guo, L. H. Hwang, Oncolytic Sindbis virus targets tumors defective in the interferon response and induces significant bystander antitumor immunity in vivo. Mol. Ther. 20, 298–305 (2012). 22068428 10.1038/mt.2011.245 PMC3277240

[43] M. P. King, G. Attardi, Human cells lacking mtDNA: Repopulation with exogenous mitochondria by complementation. Science 246, 500–503 (1989). 2814477 10.1126/science.2814477

[44] K. Hashiguchi, Q. M. Zhang-Akiyama, Establishment of human cell lines lacking mitochondrial DNA. Methods Mol. Biol. 554, 383–391 (2009). 19513686 10.1007/978-1-59745-521-3_23

