# Supplementary material for: DNA-induced 2′3′-cGAMP enhances haplotype-specific human STING cleavage by dengue protease
Source: Proc Natl Acad Sci U S A. 2020 Jun 23;117(27):15947–54. doi: 10.1073/pnas.1922243117 (PMC7354927; doi:10.1073/pnas.1922243117)
Supplement: Supplementary File [file pnas.1922243117.sapp.pdf]

PNAS
www.pnas.org

Supplementary Information for

DNA-induced 2'3'-cGAMP enhances haplotype-specific human STING cleavage by dengue protease

Chan-I Su[a,1], Yu-Ting Kao[a,1], Chao-Chen Chang[b], Yao Chang[a], Tzong-Shiann Ho[c], H. Sunny Sun[d], Yi-Ling Lin[e], Michael M. C. Lai[f,g] , Yu-Huei Liu[h] and Chia-Yi Yu[a,2]

[a]National Institute of Infectious Diseases and Vaccinology, National Health Research Institutes, Miaoli 350, Taiwan; [b]Department of Microbiology and Immunology, National Cheng Kung University, Tainan 701, Taiwan; [c]Department of Pediatrics, National Cheng Kung University, Tainan 701, Taiwan; [d]Institute of Molecular Medicine, National Cheng Kung University, Tainan 701, Taiwan; [e]Institute of Biomedical Sciences, Academia Sinica, Taipei 115, Taiwan; [f]Research Center for Emerging Viruses, China Medical University Hospital, Taichung 404, Taiwan; [g]Institute of Molecular Biology, Academia Sinica, Taipei 115, Taiwan; [h]Graduate Institute of Integrated Medicine, China Medical University, Taichung 404, Taiwan

[1]Equal contribution.

[2]Corresponding author: Chia-Yi Yu, Ph.D. Email: epitope@nhri.org.tw

This PDF file includes:

Figs. S1 to S14
Tables S1 to S4

www.pnas.org/cgi/doi/10.1073/pnas.1922243117

```
                                                                                                                   100
RGRR  ATGCCCCATTCCAGCCTGCATCCATCCATCCCGTGTCCCAGGGGTCACGG GGCCCAGAAGGCAGCCTTGGTTCTGCTGAGTGCCTGCCTGGTGACCCTTT
HARQ  .................................................. ..................................................
RGHR  .................................................. ..................................................

                                                                                                                   200
RGRR  GGGGGCTAGGAGAGCCACCAGAGCACACTCTCCGGTACCTGGTGCTCCAC CTAGCCTCCCTGCAGCTGGGACTGCTGTTAAACGGGGTCTGCAGCCTGGC
HARQ  .................................................. ..................................................
RGHR  .................................................. ..................................................

                                                                                                                   300
RGRR  TGAGGAGCTGCGCCACATCCACTCCAGGTACCGGGGCAGCTACTGGAGGA CTGTGCGGGCCTGCCTGGGCTGCCCCCTCCGCCGTGGGGCCCTGTTGCTG
HARQ  ...........A...................................... ..................................................
RGHR  .................................................. ..................................................

                                                                                                                   400
RGRR  CTGTCCATCTATTTCTACTACTCCCTCCCAAATGCGGTCGGCCCGCCCTT CACTTGGATGCTTGCCCTCCTGGGCCTCTCGCAGGCACTGAACATCCTCC
HARQ  .................................................. ..................................................
RGHR  .................................................. ..................................................

                                                                                                                   500
RGRR  TGGGCCTCAAGGGCCTGGCCCCAGCTGAGATCTCTGCAGTGTGTGAAAAA GGGAATTTCAACGTGGCCCATGGGCTGGCATGGTCATATTACATCGGATA
HARQ  .................................................. ..................................................
RGHR  .................................................. ..................................................

                                                                                                                   600
RGRR  TCTGCGGCTGATCCTGCCAGAGCTCCAGGCCCGGATTCGAACTTACAATC AGCATTACAACAACCTGCTACGGGGTGCAGTGAGCCAGCGGCTGTATATT
HARQ  .................................................. ..................................................
RGHR  .................................................. ..................................................

                                                                                                                   700
RGRR  CTCCTCCCATTGGACTGTGGGGTGCCTGATAACCTGAGTATGGCTGACCC CAACATTCGCTTCCTGGATAAACTGCCCCAGCAGACCGGTGACCGTGCCG
HARQ  .................................................. .......................................C........T.
RGHR  .................................................. ............................................A...T.

                                                                                                                   800
RGRR  GCATCAAGGATCGGGTTTACAGCAACAGCATCTATGAGCTTCTGGAGAAC GGGCAGCGGGCGGGCACCTGTGTCCTGGAGTACGCCACCCCCTTGCAGAC
HARQ  .................................................. ..................................................
RGHR  .................................................. ..................................................

                                                                                                                   900
RGRR  TTTGTTTGCCATGTCACAATACAGTCAAGCTGGCTTTAGCCGGGAGGATA GGCTTGAGCAGGCCAAACTCTTCTGCCGGACACTTGAGGACATCCTGGCA
HARQ  .................................................. ...........................A......................
RGHR  ........................................ ......... ..................................................

                                                                                                                  1000
RGRR  GATGCCCCTGAGTCTCAGAACAACTGCCGCCTCATTGCCTACCAGGAACC TGCAGATGACAGCAGCTTCTCGCTGTCCCAGGAGGTTCTCCGGCACCTGC
HARQ  .................................................. ..................................................
RGHR  .................................................. ..................................................

                                                                                                                  1100
RGRR  GGCAGGAGGAAAAGGAAGAGGTTACTGTGGGCAGCTTGAAGACCTCAGCG GTGCCCAGTACCTCCACGATGTCCCAAGAGCCTGAGCTCCTCATCAGTGG
HARQ  .................................................. ..................................................
RGHR  .................................................. ..................................................

                                        1137
RGRR  AATGGAAAAGCCCCTCCCTCTCCGCACGGATTTCTCT
HARQ  .....................................
RGHR  .....................................
```

Fig. S1. Nucleotide sequence alignment of RGRR, HARQ, and RGHR haplotypes used in this study.

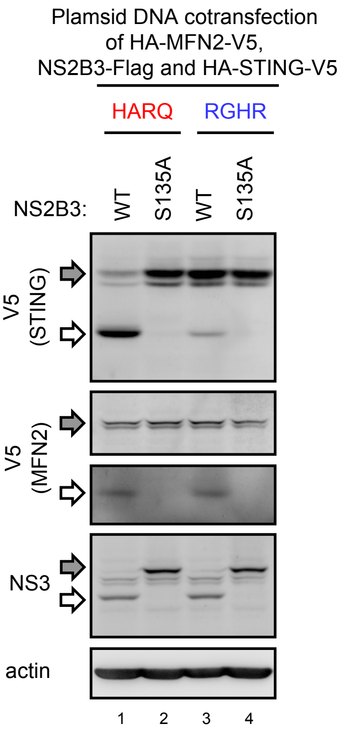

Fig. S2. Haplotype of STING, rather than the protease activity of NS2B3, affects the STING cleavage. A549 cells were cotransfected with Flag-tagged DENV protease NS2B3, V5-tagged MFN2 and each V5-tagged STING for 18 h. Cell lysates were analyzed by WB using the indicated primary antibodies. S135A, protease-dead mutant. Gray arrow, full-length STING-V5, MFN2-V5 or NS2B3; white arrow, cleaved STING-V5, MFN2-V5 or NS3.

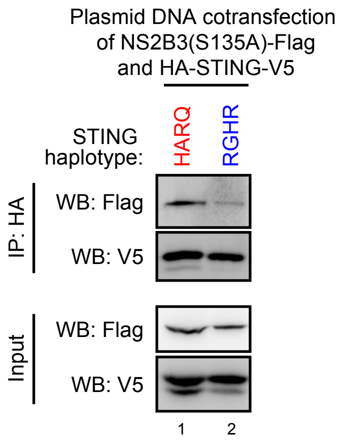

Fig. S3. Interaction between DENV protease and human STINGs. 293T/17 cells were cotransfected with NS2B3(S135A) and each STING haplotype for 24 h. The interaction was analyzed by co-immunoprecipitation (Co-IP) and WB with the indicated primary antibodies.

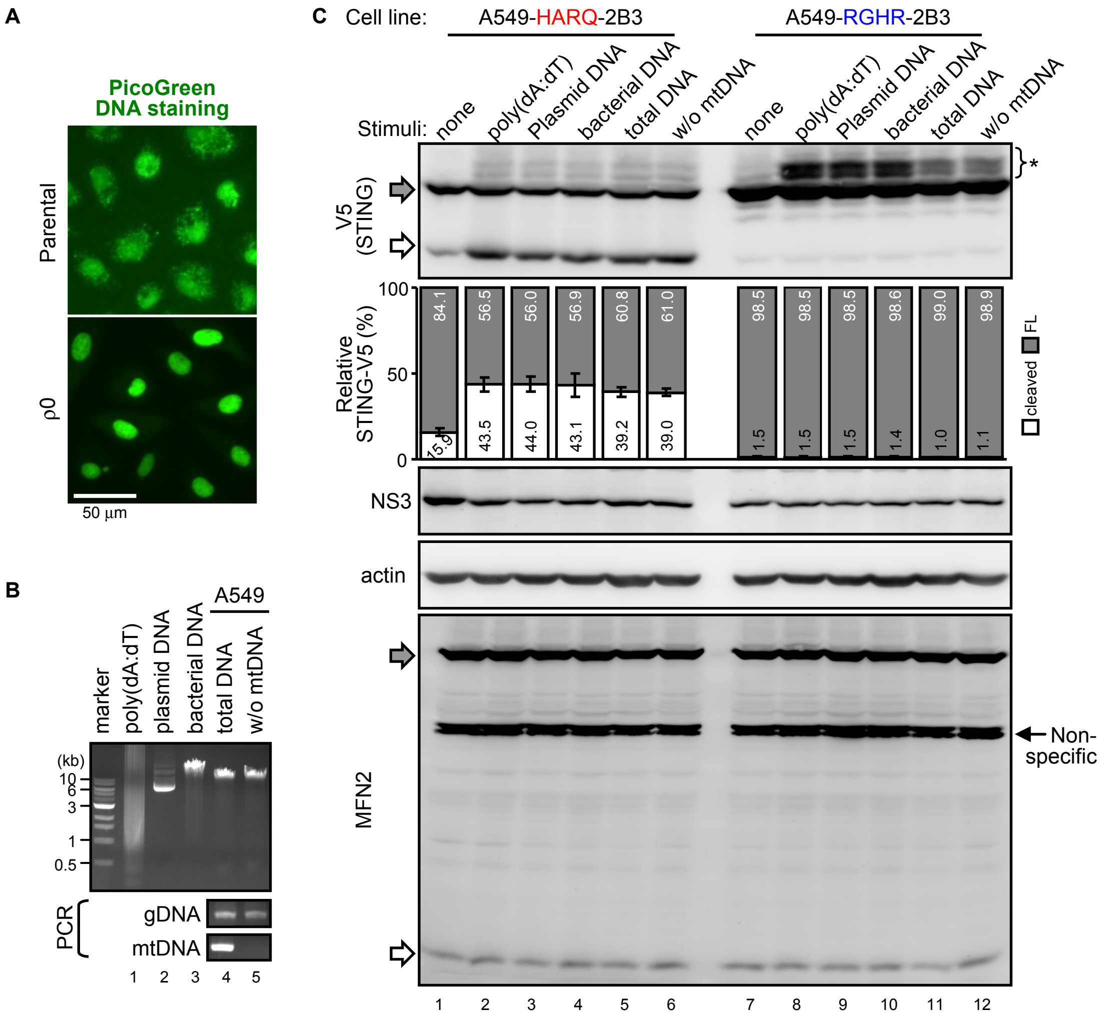

Fig. S4. DENV protease-mediated HARQ cleavage can be further enhanced by various DNA stimuli. (*A*) A549 cells were cultured with the same culture media but with (ρ0) or without (parental) EtBr (50 ng/ml) in parallel for > 6 months. The depletion of mtDNA in ρ0 cells was monitored by PicoGreen DNA staining. (*B*) DNA preparations used here were analyzed by agarose electrophoresis. DNA derived from A549 cells were further analyzed by PCR with specific primers targeting genomic DNA (gDNA) or mitochondrial DNA (mtDNA). (*C*) A549 cells stably coexpressing DENV protease (2B3) and each STING were stimulated with the indicated DNA species for 4 h, then analyzed by WB with the indicated antibodies. Poly(dA:dT), a synthetic analog of B-form DNA; plasmid DNA, the plasmid DNA Flag-GFP/pCR3.1; bacterial DNA, total DNA derived from *E. coli* competent cells; total DNA, total DNA from A549 cells; w/o mtDNA, total DNA from mtDNA-depleted A549-ρ0 cells. Gray arrow, full-length of STING-V5 or endogenous MFN2; white arrow, cleaved STING-V5 or MFN2. Quantification of the STING cleavage levels was in a bar graph. The full-length (FL; gray bar) and cleaved (white bar) STING-V5 signal were divided by the total STING-V5 signal of each lane for the relative ratio in percentage. Data are mean ± SD (n = 3 per group).

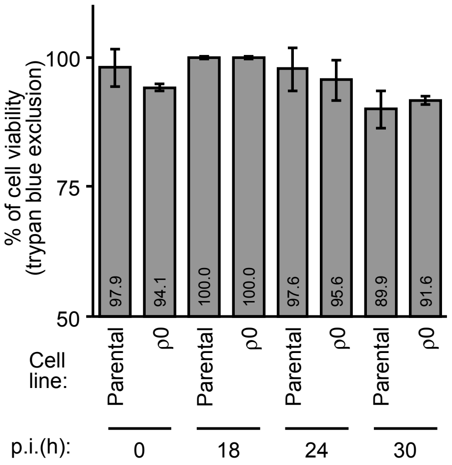

Fig. S5. Viability of A549-HARQ parental and ρ0 cells after DENV infection. A549-HARQ parental and its mtDNA-depleted ρ0 cells were infected with DENV (moi 5). The cell viability was monitored by trypan blue exclusion assay at the indicated time. p.i., post infection. Data are mean ± SD (*n* = 3 per group).

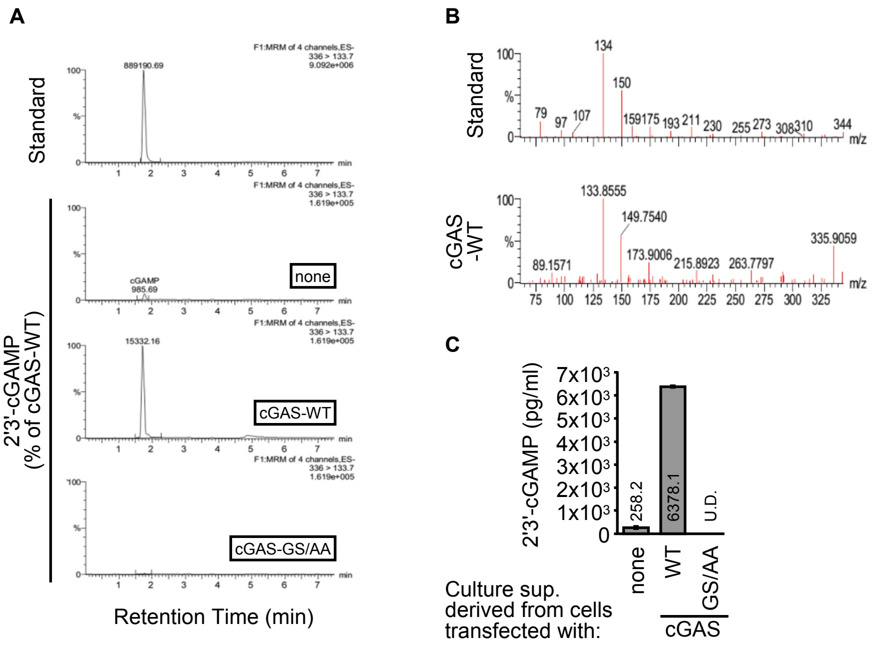

Fig. S6. Mass spectrometry-based quantification of 2'3'-cGAMP in the culture supernatant. (*A*) Chromatograms of 2'3'-cGAMP standard and indicated samples based on the MRM mode with transition m/z 336 ($[cGAMP\text{-}2H]^{2-}$) to 133.7 were shown. (*B*) Daughter ions of the sample and cGAMP standard were compared by the Product ion confirmation scanning (PICS). (*C*) Quantification of the detected 2'3'-cGAMP in the indicated groups. Data are mean ± SD (n = 3 per group). The limit of detection (LOD) of 2'3'-cGAMP in medium was 0.2132 pg/ml calculated by degree of freedom 2 and two-sided 95% confidence.

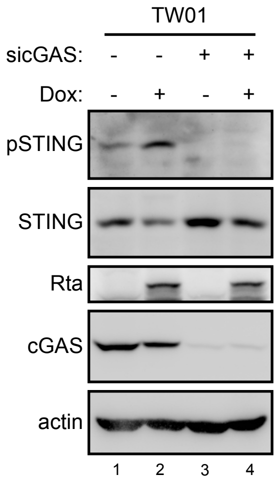

Fig. S7. Activation of cGAS–STING pathway in TW01 cells. TW01 cells were transfected with control siRNA (-) or siRNA targeting cGAS (+) for two days. Afterwards, the inducible EBV reactivation system in siRNA-treated TW01 were activated by administration of doxycycline (Dox, 0.1 μg/ml, 24 h). Cell lysates were examined by WB analysis with the indicated primary antibodies. pSTING, phosphorylated STING (active form); Rta, EBV Rta protein.

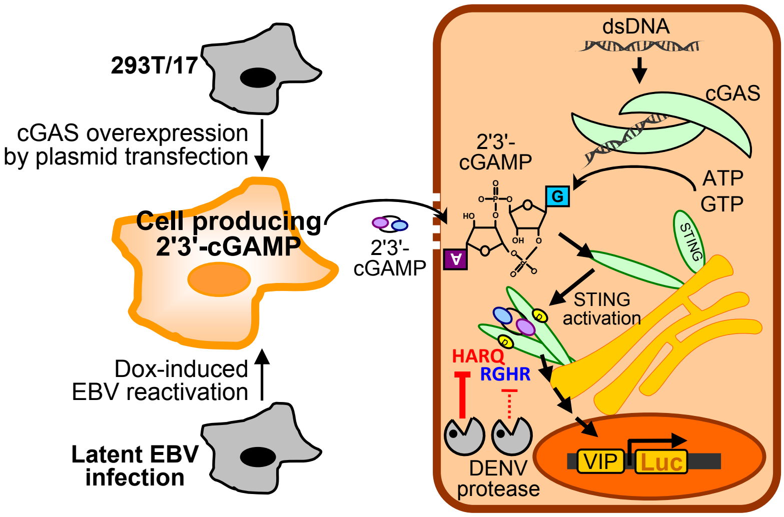

Fig. S8. Human STING haplotypes respond differently to dengue protease cleavage. Cleavage of protease-sensitive STING haplotype HARQ, but not the resistant RGHR, can be further enhanced by 2'3'-cGAMP. The enhanced DENV protease-mediated HARQ cleavage could be found by co-culture with bystander cells producing 2'3'-cGAMP, either by DNA transfection of cGAS or by reactivating Epstein–Barr virus (EBV) from latent infection.

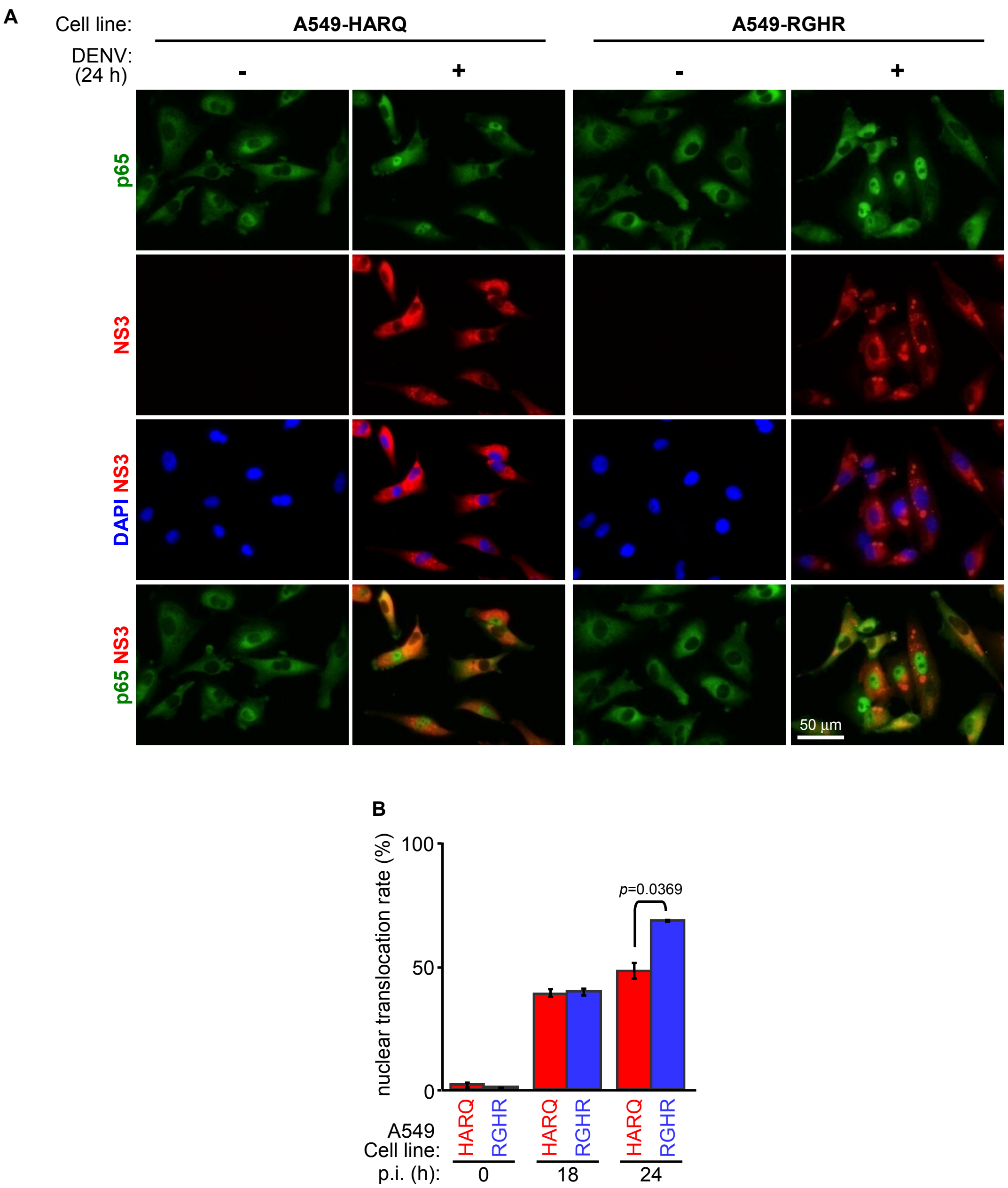

Fig. S9. Nucleus translocation of NF-κB p65 in DENV-infected cells. (*A*) A549-HARQ and A549-RGHR cells were infected with DENV (moi 10) for the indicated time and then analyzed by IFA using the indicated antibodies. (*B*) The nucleus translocation rate was shown in the bar graph. Data are mean ± SD, $n = 3$ per group.

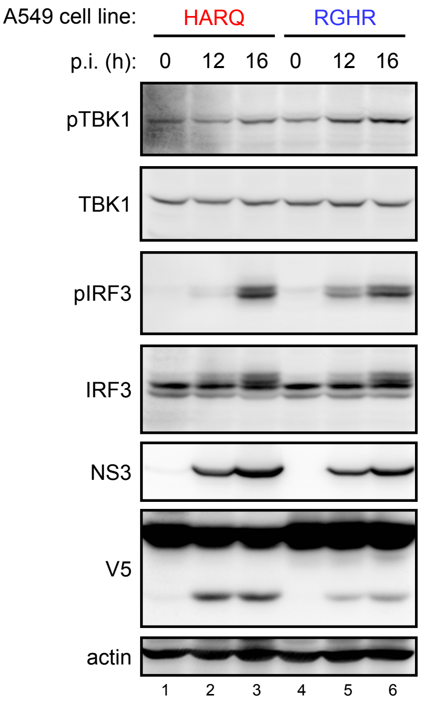

Fig. S10. Complete blot images of the Figure 4*C*.

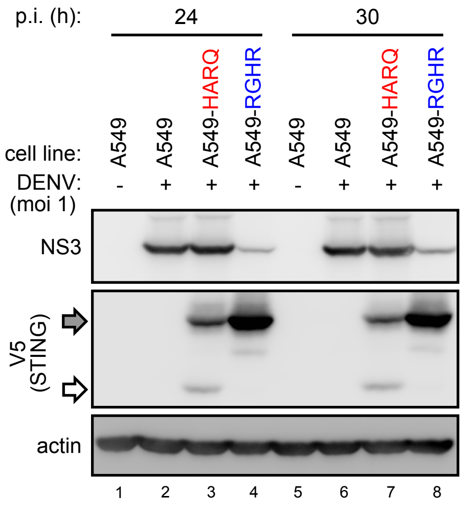

Fig. S11. Antiviral effects of human STINGs. A549-HARQ or A549-RGHR cells were infected with DENV (moi 1) for the indicated time, then analyzed by WB. Gray arrow, full-length STING-V5; white arrow, cleaved product of STING.

A

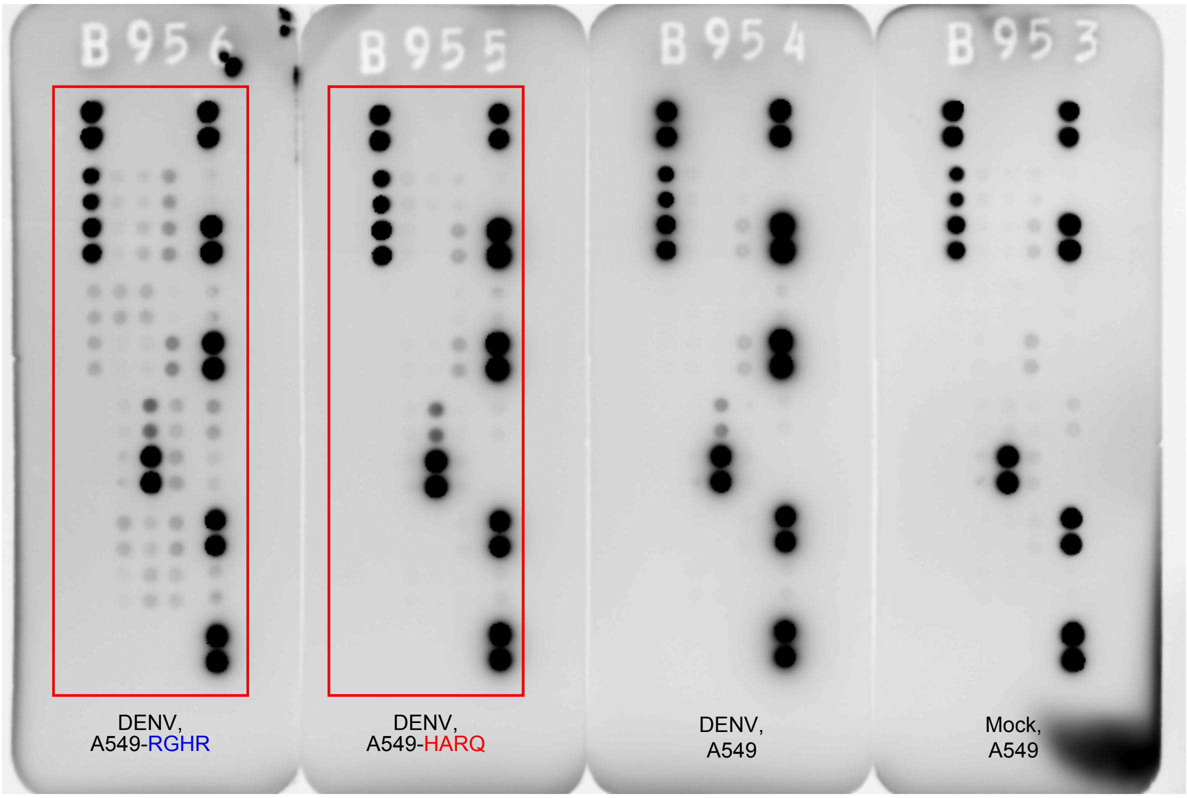

B

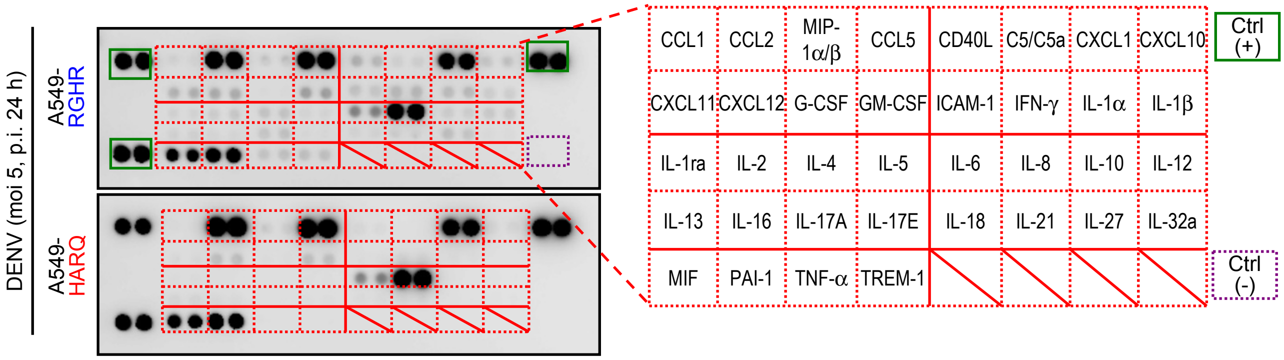

Fig. S12. Complete blot images of the Figure 4*F*. (*A*) Red boxes indicate the cropped areas shown in the manuscript. (*B*) Relative position of the human cytokine array detects 36 cytokines with duplicate spots were shown.

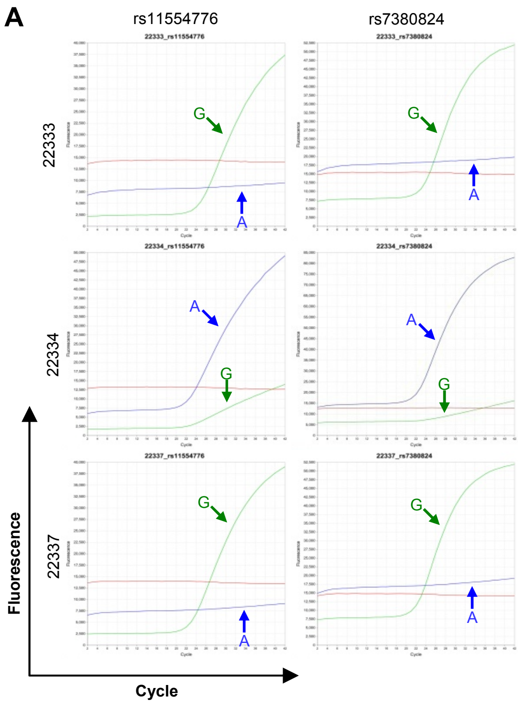

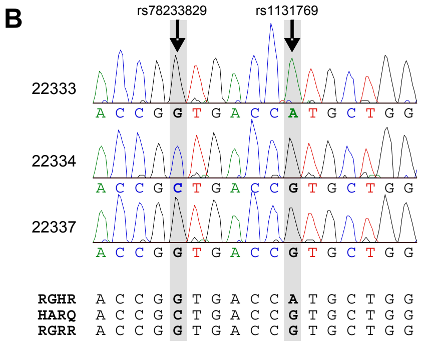

C

P't#	SNPs: Rs115-54776 (aa 71)	Rs782-33829 (aa 230)	Rs113-1769 (aa 232)	Rs738-0824 (aa 293)	Haplo-types
**22333**	GG	GG	AA	GG	RGHR/ RGHR
**22334**	AA	CC	GG	AA	HARQ/ HARQ
**22337**	GG	GG	GG	GG	RGRR/ RGRR

Fig. S13. Representative examples of STING haplotype genotyping data derived from Taiwanese people with DENV infection. The genomic DNA derived from each sample was amplified by PCR. The rs11554776 and rs7380824 were identified by qPCR using specific primer and probe sets (*A*), whereas the rs78233829 and rs1131769 were identified by DNA sequencing (*B*) as described in Methods. The genotyping results were summarized (*C*).

A

	NC	P't
RGRR	67	86
HARQ	89	74
RGHR	19	26
others	3	2
	178	188

B

n (%)	HARQ	RGRR	RGHR
HARQ	NC: 26 (29.21%)<br>P't: 14 (14.89%)		
RGRR	NC: 27 (30.34%)<br>P't: 37 (39.36%)	NC: 15 (16.85%)<br>P't: 16 (17.02%)	
RGHR	NC: 9 (10.11%)<br>P't: 7 (7.45%)	NC: 9 (10.11%)<br>P't: 17 (18.09%)	NC: 0 (0.00%)<br>P't: 1 (1.06%)
Others	NC: 3 (3.37%)<br>P't: 2 (2.13%)		

NC: Normal control, n=89
P't: Patient with DF, n=94

Fig. S14. The distribution of STING haplotypes in Taiwan population. (*A*) genomic DNA from normal control Taiwanese (NC, $n = 89$) and Taiwanese people with DENV infection (P't, $n = 94$) were analyzed for STING haplotypes. The contingency table of STING haplotype numbers in NC and P't group was shown. There are 67 RGRR, 89 HARQ, 19 RGHR, and 3 other STING haplotypes in NC group, whereas P't group contains 86 RGRR, 74 HARQ, 26 RGHR, and 2 other STINGs. (*B*) STING genotypes in NC and P't groups were shown in two-dimensional array. Total sample size: 94 NC and 102 P't; Genotyping data exclusion: 5 NC samples and 8 P't samples were excluded because of missing values in SNP identification.

Table S1. Primer sequences used in this study

Primer Name	Primer Sequence
STING(230G/232H)	AACTGCCCCAGCAGACCGGTGACCATGCTGGCATCAAGGATCGG
STING(293R)	GCAGGCCAAACTCTTCTGCCGGACACTTGAGGACATCCTGGCA
STING(71R)	GCCTGGCTGAGGAGCTGCGCCACATCCACTCCAGGTACCG
STING(RGRR)	TGCCCCAGCAGACCGCGGACCATGCTGGCATCAAGGATCGG
Asc1-HA	TTGGCGCGCCACCATGTACCCATACGATGTTCCAG
BGH pA/R	CTAGAAGGCACAGTCGAGG
hcGAS(-23--6)	TCGGGGAACAGAAAGCGC
hcGAS(1547~1573)	CAATCTCAAAATTCATCAAAAACTGGA
Xho1-hcGAS(4-20)	ACCGCTCGAGCAGCCTTGGCACGGAAA
hcGAS-Sac2(1566-1540)	TCCCCGCGGAAATTCATCAAAAACTGGAAACTCATTGT
cGAS(GS/AA)	CGGGCTGCTGAACACCGCGGCCTACTATGAGCACGTGAAGATTTCTG
EcoRI-Luc(1653-1632)	TACATGAATTCTTACACGGCGATCTTTCCGCCC
EcoRI-FLuc (1-16)	AAGCCGAATTCACCATGGAAGACGCCAAAA
mtDNA-F	CCTAGGGATAACAGCGCAAT
mtDNA-R	TAGAAGAGCGATGGTGAGAG
HuH1-F	ATGAGCTCATGACCGAGAATTCCACGTCCG
HuH1-R	ATCCCGGGCAAACTTCTTCTTGCC
hIFNb-F	CACGACAGCTCTTTCCATGA
hIFNb-R	AGCCAGTGCTCGATGAATCT
hCXCL10-F	CTGAGACATTCCTCAATTGCTTAGAC
hCXCL10-R	AGGCAGCCTCTGTGTGGTCCATCCT
Actin-F	TCCTGTGGCATCCACGAAACT
Actin-R	GAAGCATTTGCGGTGGACGAT
TMEM173-F	GACCTGGGTCTCACTCCTGA
TMEM173-R	AGCTCAGAGAAGGGCAGTGA

Table S2. Sources of cell lines used in this study

Cell lines	Sources
A549	Yu, C. Y. *et al. Plos Pathog* **8**, e1002780 (2012).
A549-2B3(S135A)	Yu, C. Y. *et al. Plos Pathog* **8**, e1002780 (2012).
A549-HARQ-2B3	This paper
A549-RGHR-2B3	This paper
A549-HARQ-2B3-Vip-Luc	This paper
A549-RGHR-2B3-Vip-Luc	This paper
A549-ρ0	This paper
A549-HARQ-Parental	This paper
A549-HARQ-ρ0	This paper
293T/17	ATCC CRL-11268
Vero	ATCC CCL-81
TW01-ERGV62	Lan, Y. Y. *et al. Plos One* **8**, e56121 (2013).

Table S3. Antibodies used in this study

Antibodies	Sources
NS3 (AS3-274)	Yu, C. Y. *et al. Plos Pathog* **8**, e1002780 (2012).
Dengue virus NS3 protein antibody [GT2811]	GeneTex GTX629477; RRID:AB_2801283
NS3 (Dengue virus 2) antibody	GeneTex GTX124252; RRID:AB_11171668
Rta (467)	Lan, Y. Y. *et al. Plos One* **8**, e56121 (2013).
DNA antibody [AC-30-10]	GeneTex GTX41384; RRID:AB_11174631
cGAS (D1D3G) antibody	Cell Signaling Technology #15102; RRID: AB_2732795
Phospho-STING (Ser366) Antibody	Cell Signaling Technology #85735; ; RRID:AB_2801279
STING (D2P2F) antibody	Cell Signaling Technology #13647; RRID:AB_2732796
beta Actin antibody [GT5512]	GeneTex GTX629630; RRID: AB_2728646
V5-Tag (D3H8Q) Rabbit Antibody	Cell Signaling Technology #13202; RRID:AB_2687461
V5 tag antibody [sv5-pk1]	GeneTex GTX42525; RRID:AB_510174
Rabbit Anti-HA-Tag Monoclonal Antibody, Unconjugated, Clone C29F4	Cell Signaling Technology #3724s; RRID:AB_1549585
Monoclonal ANTI-FLAG® M2 antibody	Sigma-Aldrich F1804; RRID:AB_262044
TBK1/NAK Antibody	Cell Signaling Technology Cat# 3013, RRID:AB_2199749
Phospho-TBK1/NAK (Ser172) (D52C2) XP Rabbit monoclonal Antibody	Cell Signaling Technology Cat# 5483, RRID:AB_10693472
IRF-3 (SL-12) antibody	Santa Cruz Biotechnology Cat# sc-33641, RRID:AB_627826
IRF3 (phospho S386) antibody [EPR2346]	Abcam Cat# ab76493, RRID:AB_1523836
Mfn2 (XX-1) antibody	Santa Cruz Biotechnology Cat# sc-100560, RRID:AB_2235195
Goat Anti-NF kappa B p65 Polyclonal antibody, Unconjugated	Santa Cruz Biotechnology Cat# sc-372, RRID:AB_632037
Peroxidase-AffiniPure Goat Anti-Rabbit IgG (H+L) (min X Hu,Ms,Rat Sr Prot) antibody	Jackson ImmunoResearch Labs 111-035-144; RRID: AB_2307391
Peroxidase-AffiniPure Goat Anti-Mouse IgG (H+L) (min X Hu,Bov,Hrs,Rb,Sw Sr Prot) antibody	Jackson ImmunoResearch Labs 111-035-146; RRID: AB_2307392
Goat anti-Mouse IgG (H+L) Cross-Adsorbed Secondary Antibody, Alexa Fluor 568	Invitrogen # A-11004; RRID:AB_2534072
Goat anti-Rabbit IgG (H+L) Cross-Adsorbed Secondary Antibody, Alexa Fluor 488	Invitrogen # A-11008; RRID: AB_143165
Goat anti-Mouse IgM Heavy Chain Cross-Adsorbed Secondary Antibody, Alexa Fluor 488	Invitrogen A-21042; RRID: AB_2535711

Table S4. A list of nucleic acid stimuli used in this study

Figure	Nucleic acid	Concentration
Fig. 1*D*	Poly(dA:dT)	1 µg/ml
Fig. 1*E*	Poly(dA:dT)	1 µg/ml
Fig. 2*D*	Poly(dA:dT)	1 µg/ml
Fig. 3*B*	Poly(dA:dT)	1 µg/ml
	2'3'-cGAMP	10 µg/ml
	3'3'-cGAMP	10 µg/ml
	c-diGMP	10 µg/ml
	c-diAMP	10 µg/ml
Fig. S4*C*	Poly(dA:dT)	1 µg/ml
